# Risk of recurrence of retinopathy of prematurity after initial intravitreal ranibizumab therapy

**DOI:** 10.1038/srep27082

**Published:** 2016-06-01

**Authors:** Joyce J. T. Chan, Carol P. S. Lam, Madeline K. M. Kwok, Raymond L. M. Wong, Gary K. Y. Lee, Winnie W. Y. Lau, Jason C. S. Yam

**Affiliations:** 1 Department of Ophthalmology and Visual Sciences, Hong Kong Eye Hospital, 147K, Argyle Street, Kowloon City, Kowloon, Hong Kong SAR; 2 Department of Ophthalmology and Visual Sciences, The Chinese University of Hong Kong, Hong Kong Eye Hospital, 147K, Argyle Street, Kowloon City, Kowloon, Hong Kong SAR

## Abstract

We report our experience with the use of intravitreal ranibizumab for the treatment of retinopathy of prematurity (ROP). A retrospective review was performed on 138 consecutive infants screened at a single centre over 18 months. Intravitreal ranibizumab was offered in selected cases requiring treatment, such as aggressive posterior ROP or poor mydriasis. 2 eyes of 1 infant received intravitreal ranibizumab alone and 8 eyes of 5 infants received combined intravitreal ranibizumab and laser therapy. 3 out of 8 eyes treated initially with intravitreal ranibizumab monotherapy had persistent disease requiring laser therapy, and 3 out of 5 eyes with initial regression suffered disease recurrence at a mean of 7.6 weeks post-injection. 2 eyes treated first with laser followed by intravitreal ranibizumab had disease regression without recurrence. Our cohort demonstrate a significant rate of persistent disease and recurrence in ROP eyes treated initially with intravitreal ranibizumab monotherapy, which is greater and earlier than that reported for intravitreal bevacizumab in the BEAT-ROP study. Intravitreal ranibizumab may be useful as an initial treatment in selected cases of ROP when laser therapy as first line is suboptimal. However, close monitoring is important and adjunctive laser therapy may subsequently be needed in a majority of cases.

Retinopathy of prematurity (ROP) is caused by abnormal development of retinal vessels in the immature retinas of preterm infants with very low birth weight. In 2010, an estimated 20,000 infants became blind or severely visually impaired from ROP worldwide^1^. ROP remains a leading cause of childhood blindness as advances in neonatal intensive care improve the survival of very immature infants at high risk for the disease. Risk factors for ROP include low gestational age and birth weight^2^, early high and late low oxygen saturation^3^, early post-natal low serum insulin-growth factor-1 concentration^4^, hyperglycemia^5^ and neonatal infections^6^, and it often occurs in conjunction with co-morbidities such as bronchopulmonary dysplasia, cerebral white matter damage and necrotizing enterocolitis^7^. Local studies on Chinese infants in Hong Kong^8^,^9^ showed a 14.5% and 3.9% risk of developing Type 1 ROP in extreme low birth weight infants of less than 1000 g and infants of multiple gestations respectively. In preterm Chinese infants, younger gestational age, lower birth weight, postnatal hypotension, inotrope use, bronchopulmonary dysplasia and intraventricular haemorrhage were independent risk factors for the development of ROP.

ROP is a biphasic condition characterized by an initial phase of hyperoxia and loss of maternal-foetal interaction resulting in an arrest of retinal vascularization, followed by a second phase of hypoxia-induced aberrant vasoproliferation in an increasingly metabolically active yet poorly vascularized retina^10^. In most infants ROP regresses spontaneously. However, in a small proportion of infants, abnormal retinal neovascularization progresses aggressively to fibrous scarring, distortion, tractional retinal detachment, and ultimately blindness if left untreated. The current standard of care for ROP involves peripheral retinal laser photocoagulation for Type 1 ROP, based on the results of the Early Treatment of Retinopathy of Prematurity (ETROP) trial^11^. Laser therapy has largely replaced treatment with cryotherapy^12^ and aims at destruction of the peripheral avascular retina to induce regression of neovascularization. More recently, however, intravitreal injection of anti-vascular endothelial growth factor (anti-VEGF) has also been used as an alternative treatment for ROP. Vascular endothelial growth factor plays an important role in stimulating retinal neovascularization and elevated levels have been detected in eyes with ROP^13^,^14^,^15^. The most commonly reported anti-VEGF agent, bevacizumab, has been shown to have significant benefit in treating ROP in zone I, but not posterior zone II, stage 3 plus disease when compared with conventional laser therapy in the BEAT-ROP trial^16^. However, results from the trial also demonstrated a 4% risk of delayed recurrence at a mean of 16 weeks post-injection of bevacizumab, suggesting that close monitoring is required until complete vascularization of the retina has occurred.

Advantages of intravitreal anti-VEGF therapy compared with conventional laser therapy include reduced treatment time, avoidance of a general anaesthesia, suitability in infants with poor fundal view due to anterior segment involvement, which avoids ablation of and allows further vascularization of the peripheral retina especially in posterior disease, and possibly better refractive outcomes^17^,^18^. However, the ideal dosing, optimal technique of injection, risk of reactivation and long-term systemic safety are still unknown and remain important concerns associated with the use of intravitreal anti-VEGF therapy for ROP. Ranibizumab is an alternative anti-VEGF agent with a shorter half-life^19^,^20^,^21^ and potentially less systemic toxicity. Its use in the treatment of ROP has been reported recently in several case reports and series with variable results^22^,^23^,^24^,^25^,^26^,^27^,^28^,^29^. We report our experience with the use of intravitreal ranibizumab, in combination with laser therapy if necessary, for the treatment of ROP.

## Methods

A retrospective chart review was performed on consecutive infants screened for ROP at Queen Elizabeth Hospital, Hong Kong from July 2012 to December 2013. According to the American Academy of Paediatrics guidelines published in 2013^30^, infants born at or below 30 weeks of gestational age (GA) who had a birth weight of less than 1500 g, or those with an unstable clinical course and deemed high risk by their attending neonatologist, were screened. Age at initial examination was at post-menstrual age (PMA) of 31 weeks or 4 weeks after birth for those born at a GA of 28 weeks or more. Mydriatic fundal examination using binocular indirect ophthalmoscopy was used for all screenings. Treatment was performed in all cases of Type 1 ROP (defined as zone I: stage 3, or stage 1 or 2 with plus disease; or zone II: stage 2 or 3 with plus disease)^11^ or worse. Conventional laser therapy was the treatment of choice, but intravitreal anti-VEGF was offered in selected cases, such as aggressive posterior retinopathy of prematurity (APROP), and cases with poor pupil dilation and/or media opacity whereby laser therapy could not be performed. Since bevacizumab was not approved for intraocular use in our hospital, ranibizumab was the anti-VEGF agent of choice. In accordance to the International Classification of Retinopathy of Prematurity, APROP was defined by its posterior location (zone 1 or posterior zone 2), prominence of plus disease, modest-appearing peripheral retinopathy and rapid deterioration that may not progress through the classic stages^31^. All treated infants were examined until total regression of ROP and full vascularization. Laser therapy was performed if possible when there was persistent disease or recurrence.

Intravitreal ranibizumab injection was performed as follows. Written informed consent was obtained from the parents. A topical anaesthetic was applied, standard aseptic eye preparation with 5% betadine was performed, a lid speculum was inserted and intravitreal injection of 0.25 mg/0.025 ml ranibizumab with a 30-gauge needle was given. In one infant, bilateral injections were performed 0.5 mm post-limbus, which resulted in cataract formation in one eye. In all other infants, injections were performed 1 mm post-limbus to avoid this complication. Central artery perfusion was confirmed after the injection. Infants were examined 1 day after the procedure and regularly thereafter according to their clinical condition until regression of ROP and full vascularization of the retina was observed.

Data collection and analysis were performed using Microsoft Excel (Redmond, WA) and PASW Statistics for Windows 18.0 (SPSS Inc., Chicago). The data was summarized using descriptive statistics, and the Mann-Whitney test and Fisher’s exact test were used to test the differences between infants who received different treatments and those with different outcomes.

The study was conducted with Institutional Review Board approval and adhered to the tenets of the Declaration of Helsinki.

## Results

138 infants were screened over an 18-month period. 18 eyes of 9 infants received treatment for ROP. (see Table 1) The mean gestational age and birth weight of all infants who received treatment for ROP were 24 weeks 6 days (SD 1.8 weeks) and 687.5 g (SD 236.1 g) respectively. These infants had multiple systemic co-morbidities, including necrotizing enterocolitis (33%), sepsis (100%), bronchopulmonary dysplasia (89%) and patent ductus arteriosus (67%), requiring blood transfusion (100%) and oxygen therapy (100%).

Out of the 18 eyes of 9 infants that received treatment, 2 eyes of 1 infant received intravitreal ranibizumab alone, 8 eyes of 5 infants received combined intravitreal ranibizumab and laser therapy, and 8 eyes of 5 infants received laser therapy alone. Among those that received combined therapy, 6 eyes of 3 infants received intravitreal ranibizumab first (mean PMA 36 weeks 2 days, SD 1.9 weeks), followed by laser therapy due to either persistent or recurrent disease. 2 eyes of 2 infants received laser therapy first followed by intravitreal ranibizumab injection (at PMA 39 weeks 3 days and 50 weeks 1 day). In one eye, persistent iris vessels and a poorly dilated pupil prevented supplementary laser therapy. In the other eye, there was development of subretinal fluid that hindered supplementary laser therapy.

The indications for intravitreal ranibizumab injection were APROP in 4 eyes of 2 infants, persistent iris vessels and poor pupil dilation in 5 eyes of 3 infants, and recurrence of disease after laser therapy with development of subretinal fluid that hindered supplementary laser therapy in 1 eye.

Among the 8 eyes of 4 patients that received intravitreal ranibizumab as initial monotherapy, 5 (62.5%) had initial disease regression while 3 (37.5%) had persistent disease requiring adjunctive laser therapy. One eye with APROP had persistent disease progression after injection, which later developed retinal detachment despite repeated laser therapy and a second intravitreal ranibizumab injection. The parents opted for conservative management and declined vitreoretinal surgery. One infant with bilateral poor pupil dilation had bilateral persistent disease after intravitreal ranibizumab. After the injection bilateral pupil dilatation improved, which allowed subsequent laser therapy in both eyes. In one eye, ROP regressed with no further recurrence. However, in the other eye, persistent vitreous haemorrhage affected the accurate assessment of ROP staging. When the infant’s systemic condition permitted, lensectomy and limbal-based 25G vitrectomy was performed and he was found to have closed funnel total retinal detachment. Out of the 5 eyes with initial regression, 2 (40%) had no disease recurrence while 3 (60%) suffered recurrence, ranging from 32 to 62 days (mean = 52 days) after injection, at the PMA of 38 weeks 6 days to 47 weeks (mean = 44 weeks 2 days). These eyes received one to three sessions of laser therapy, after which their ROP all regressed with good outcome. There was no statistically significant difference in gestational age or birth weight (p-values of 0.5 and 1.0 respectively) between the infant with no recurrence versus those who had recurrent or persistent disease. The 2 eyes of 2 infants that received laser therapy first followed by intravitreal ranibizumab injection both had regression of disease with no recurrence.

In the end, 8 out of 10 (80%) eyes that received ranibizumab therapy alone or in combination with laser therapy had resolution of ROP, while 6 out of 8 (75%) eyes that received laser therapy alone had resolution of ROP. 2 out of 10 eyes in the first group and 2 out of 8 eyes in the second group developed progressive disease with retinal detachment. There was no statistically significant difference in the outcome between the two groups (p-value = 1.0). The mean gestational age and birth weight of patients who received intravitreal ranibizumab were 24 weeks and 576.7 g respectively, and those of patients who received laser therapy alone were 26 weeks 5 days and 1020 g respectively. Younger gestational age and lower birth weight were significantly correlated with the use of intravitreal ranibizumab compared with laser therapy alone (p-values of 0.024 and 0.024) respectively. Patients who received intravitreal ranibizumab as their first treatment for ROP also tended to be at a younger gestational age at the time of first treatment. The mean gestational age at first treatment was 36 weeks 2 days for those who received intravitreal ranibizumab as their initial treatment, compared with 38 weeks 5 days for those who received laser therapy as an initial treatment (p-value = 0.05).

In terms of complications, one eye developed inferotemporal peripheral lens opacity not affecting the visual axis after intravitreal injection, which has remained non-progressive up to the latest follow-up at the age of 2 years and 6 months. In this eye, intravitreal injection was performed 0.5 mm post-limbus. In all subsequent eyes intravitreal injection was performed 1 mm post-limbus with no complications encountered. There were no systemic complications of intravitreal ranibizumab injection noted. Mean follow-up period was 21.6 months (range 9–36 months).

## Discussion

Results from our study demonstrate a significant rate of persistent disease as well as recurrence after regression in ROP eyes treated initially with intravitreal ranibizumab monotherapy. Intravitreal ranibizumab may be useful as an initial treatment option in challenging cases of ROP, such as APROP, poor pupil dilation or media opacity when conventional laser therapy is suboptimal or not possible. However, close monitoring is important and adjunctive laser therapy is needed subsequently in a majority of cases. In our study, 3 out of 8 eyes treated initially with intravitreal ranibizumab monotherapy had persistent disease requiring adjunctive laser therapy, and 3 out of 5 eyes with initial regression suffered disease recurrence at a mean of 7 weeks 4 days post-injection. One eye developed localized peripheral lens opacity after injection at 0.5 mm posterior to limbus. There were no other ocular or systemic complications of intravitreal ranibizumab injection. Younger gestational age, lower birth weight and younger age at initial treatment were significantly correlated with the use of intravitreal ranibizumab versus laser therapy alone in our series. Despite the younger gestational age, lower birth weight and more aggressive disease in the ranibizumab group versus laser alone group, there was no statistically significant difference in final outcome. Possibly due to the small number of cases, we did not detect any statistically significant difference in gestational age or birth weight between those with and without disease recurrence after initial ranibizumab injection.

The use of intravitreal ranibizumab for the treatment of ROP has been described in several case reports and series with variable results. These are summarized in Table 2. Castellanos *et al.* reported 6 eyes of 3 premature infants who received intravitreal ranibizumab alone^22^. They reported excellent results, with complete resolution of neovascularization after a single injection in all eyes, continuation of normal vascular growth into the peripheral retina, no early or late recurrence, and no systemic or ocular adverse effects. However, the exact zone and stage of disease were not specified. Chen *et al.*^23^ reported similarly encouraging results of 72 eyes of 37 patients that received intravitreal injections of either bevacizumab (41 eyes of 21 patients) or ranibizumab (31 eyes of 16 patients) as the primary treatment for Type 1 ROP. 10 eyes of 5 patients had Zone 1 Stage 3 ROP, and 62 eyes of 32 patients had Zone 2 Stage 3 ROP with plus disease. All but 1 eye in the bevacizumab group had disease regression and there was no recurrence in either group after an initial good response. Our results cannot be directly compared to these two studies, however, as in our series only patients with more aggressive forms of ROP were offered ranibizumab injection.

Other studies have reported instances of disease recurrence after initial regression following ranibizumab injection in patients with more aggressive ROP. Wong *et al.* reported ten eyes from six infants that received intravitreal anti-VEGF treatment^29^. Intravitreal anti-VEGF was offered in Zone 1 or posterior Zone 2 cases. All ten demonstrated initial regression of ROP. However, ROP reactivation occurred in five out of 6 eyes treated with ranibizumab, on average 5.9 weeks after treatment, whereas none of the four eyes treatment with bevacizumab experienced reactivation, which was statistically significant (p-value < 0.05). All six eyes treated with ranibizumab and two eyes treated with bevacizumab eventually required supplemental laser therapy for reactivation or persistent Zone III avascularity. One infant who received a unilateral injection of ranibizumab demonstrated bilateral regression of ROP. Another retrospective series on the use of intravitreal anti-VEGF for the treatment of type 1 ROP also demonstrated higher recurrence rate with ranibizumab when compared with bevacizumab. 6 out of 15 eyes that received intravitreal ranibizumab required laser photocoagulation for disease recurrence, compared with 2 of 21 eyes that received intravitreal bevacizumab^24^.

Mota *et al.* described two cases of APROP treated with combined therapy including intravitreal ranibizumab and laser photocoagulation^27^. The first case was a premature infant with bilateral stage 3 zone I ROP with plus disease, bilateral tunica vasculosa lentis, pupillary rigidity, and intraretinal haemorrhages and extensive subhyaloid haemorrhage in the left eye on first screening examination. 1 week after bilateral intravitreal ranibizumab injection, there was complete regression of anterior segment vessels, reduction in central vascular dilation and tortuosity and decrease in haemorrhagic collections. Laser photocoagulation was performed 1 week after the injection. There was recurrence of disease 4 weeks later with a new subhyaloid haemorrhage in the left eye and persistence of some intraretinal haemorrhages in the right eye. A second bilateral injection of ranibizumab and supplementary laser photocoagulation was done in both eyes. Total regression was noted thereafter. The second case had bilateral stage 3 zone 1 ROP with plus disease at first screening. Laser photocoagulation was performed. One week after treatment, there was progression of the disease to stage 4A. Ranibizumab injection and supplementary laser photocoagulation was performed bilaterally. Two weeks after combined treatment, regression of neovascularization was observed. Lin *et al.*^26^ reported a case of zone 1 stage 3 ROP with plus disease. She was treated with bilateral intravitreal bevacizumab injection and neovascularization regressed for 2 months. However, zone 1 stage 3 disease with plus recurred and dense laser photocoagulation was performed. One month after the laser treatment, the NV persisted without tractional retinal detachment. Intravitreal ranibizumab injection was performed and fundus examination one month after showed complete regression of extraretinal fibrovascular proliferation. Similar to our results, these reports suggest that combined treatment with intravitreal anti-VEGF and laser therapy is often required in the management of APROP. Jang *et al.*^28^ reported a patient with bilateral zone 1 stage 3 plus ROP who was treated with combined laser photocoagulation and ranibizumab. There was initial regression but bilateral retinal detachment occurred 4 months after injection, illustrating the risk of delayed recurrence after intravitreal ranibizumab.

In our study, one eye developed progressive disease with subretinal exudation after laser therapy. Supplementary laser therapy and intravitreal ranibizumab were performed to control the vascular component, as at that point there was no significant tractional component. In this case, the subretinal fluid resolved after the injection. However, we would like to note that caution is needed when considering the use of intravitreal anti-VEGF in advanced ROP especially when there is membrane proliferation. Honda *et al.*^32^ reported a patient with acute contraction of the proliferative membrane that resulted in funnel-like retinal detachment after intravitreal bevacizumab for stage 4A ROP. This is postulated to be due to rapid neovascular involution with accelerated fibrosis in response to decreased levels of VEGF. In addition, we report a case of non-progressive peripheral lens opacity following intravitreal injection at 0.5 mm post-limbus in a premature infant. In our series, no injection-related complications were reported in cases where the injection was performed 1 mm post-limbus. The injection site for intravitreal injections in premature infants is not as well defined as in adults, and various injection sites ranging from 0.5 mm^33^ to 2.5 mm^16^ post-limbus have been reported. We did not choose 2.5 mm post-limbus, as this is trans-retinal with a possible risk of causing retinal breaks and detachment. Our results indicate that intravitreal injection at 1 mm post limbus may be safer than 0.5 mm post-limbus in premature infants.

No systemic complications following intravitreal anti-VEGF injection for ROP have been reported to date. Theoretically, systemic blockade of VEGF may result in long-term effects on the development of premature infants. Hoerster *et al.*^25^ reported a case of bilateral stage 3 plus disease at the border of zone I and II who received bilateral intravitreal ranibizumab injection. They demonstrated that systemic VEGF levels were suppressed after the injection, reaching a nadir at 2–3 weeks and returning to normal by 4 weeks post injection. They postulated that this suppression may be due to an impaired blood retinal barrier at this age as ranibizumab does not change systemic VEGF levels in adults^19^,^20^,^21^. This is in contrast to studies that have shown that intravitreal bevacizumab led to reduction in systemic VEGF in infants for almost 2 months^34^,^35^,^36^. Due to this differential effect on systemic VEGF levels, the use of intravitreal ranibizumab is postulated to be safer than bevacizumab in infants. However, some retrospective non-randomized studies mentioned above have shown a higher risk of disease recurrence after intravitreal ranibizumab when compared to intravitreal bevacizumab^24^,^29^. Compared to the BEAT-ROP study on intravitreal bevacizumab that demonstrated a 4% risk of recurrence at a mean of 16 weeks post-injection^16^, our results on intravitreal ranibizumab also seem to indicate a higher risk of recurrence (60%) at an earlier time point (mean 7 weeks 4 days). Studies have shown that VEGF is not the only growth factor that is up-regulated in ROP^13^,^14^,^37^. This may explain why inhibition of VEGF alone does not induce regression in all cases of ROP and that disease recurrence requiring supplementary laser treatment may be needed. The shorter half-life^19^,^20^,^21^ of intravitreal ranibizumab may result in a higher and earlier risk of recurrence when compared with intravitreal bevacizumab. Randomized controlled trials comparing intravitreal ranibizumab and bevacizumab are needed to determine if there are any differences in clinical efficacy, rate of reactivation as well as long-term safety in infants.

Similar to previous reports on the subject, limitations of our study include its retrospective nature, data from a single institution and small sample size. Our results also cannot be generalized to all eyes with type 1 ROP, as only eyes with more aggressive forms of ROP whereby laser would be suboptimal or not possible were offered intravitreal ranibizumab in our series.

In conclusion, our findings suggest that intravitreal ranibizumab injection as initial monotherapy for ROP is associated with a significant risk of recurrence, which is higher than that reported in the BEAT-ROP study on bevacizumab and in line with some of the other published series on ranibizumab. It is useful as an initial therapy in selected cases of ROP, such as APROP, poor pupil dilation and media opacity when conventional laser therapy cannot be effectively performed. However, close monitoring after injection is important and we suggest weekly review to actively look for recurrence. Subsequent laser treatment is needed in the majority of cases.

## Additional Information

**How to cite this article**: Chan, J. J. T. *et al.* Risk of recurrence of retinopathy of prematurity after initial intravitreal ranibizumab therapy. *Sci. Rep.* **6**, 27082; doi: 10.1038/srep27082 (2016).

## Figures and Tables

**Table 1:** Characteristics of patients who have received treatment for retinopathy of prematurity at a single centre during a study period of 1.5 years.

Case	GA	BW	NEC	Sepsis	BT	BPD	PDA	O_2_	1st treatment received	Intra-vitreal ranibizu-mab	Laser	Surgery	Stage at first injection (or laser*)	GA at first injection (or laser*)	Initial regression	Recurrence	GA at recurrence	Subsequent treatment	Outcome (stage)	FU
1 OD	24	540	N	Y	Y	Y	N	Y	Ranibizumab	Y	Y	N	APROP	34 + 2	Y	Y	38 + 6	Laser	0	19
1 OS	24	540	N	Y	Y	Y	N	Y	Ranibizumab	Y	Y	N	APROP	34 + 2	N	–	–	Laser, 2^nd^ ranibizumab	5	19
2 OD	23 + 4	460	N	Y	Y	Y	Y	Y	Laser	N	Y	N	Z2S2+	38 + 3	Y	N	–	–	0	25
2 OS	23 + 4	460	N	Y	Y	Y	Y	Y	Laser	Y	Y	N	Z2S3+ ; small pupil	39 + 3	Y	N	–	–	0	25
3 OD	23 + 4	605	Y	Y	Y	Y	Y	Y	Ranibizumab	Y	N	N	APROP	34 + 5	Y	N	–	–	0	28
3 OS	23 + 4	605	Y	Y	Y	Y	Y	Y	Ranibizumab	Y	N	N	APROP	34 + 5	Y	N	–	–	0	28
4 OD	24	630	N	Y	Y	Y	Y	Y	Laser	N	Y	N	Z2S3+	39 + 5	Y	N	–	–	0	14
4 OS	24	630	N	Y	Y	Y	Y	Y	Laser	Y	Y	N	Z2S4a	50 + 1	Y	N	–	–	0	14
5 OD	24 + 6	720	N	Y	Y	Y	Y	Y	Ranibizumab	Y	Y	N	Z2S3+ ; small pupil	38 + 1	Y	Y	47	Laser	0	31
5 OS	24 + 6	720	N	Y	Y	Y	Y	Y	Ranibizumab	Y	Y	N	Z2S3+ ; small pupil	38 + 1	Y	Y	47	Laser	0	31
6 OD	24 + 3	505	Y	Y	Y	Y	N	Y	Ranibizumab	Y	Y	N	Z2S3+ ; small pupil	38	N	–	–	Laser	0	21
6 OS	24 + 3	505	Y	Y	Y	Y	N	Y	Ranibizumab	Y	Y	Y	Z2S3+ ; small pupil	38	N	–	–	Laser, Surgery	5	21
7 OD	25 + 3	801	N	Y	Y	Y	Y	Y	Laser	N	Y	N	Z2S2+	39 + 6	Y	N	–	–	0	9
7 OS	25 + 3	801	N	Y	Y	Y	Y	Y	Laser	N	Y	N	Z2S2+	39 + 6	Y	N	–	–	0	9
8 OD	25 + 3	860	Y	Y	Y	Y	Y	Y	Laser	N	Y	N	Z2S3+	41 + 6	Y	N	–	–	0	36
8 OS	25 + 3	860	Y	Y	Y	Y	Y	Y	Laser	N	Y	N	Z2S3+	41 + 6	Y	N	–	–	0	36
9 OD	29 + 3	1180	N	Y	Y	N	N	Y	Laser	N	Y	Y	Z2S3+	37 + 6	N	–	–	Laser, surgery	4b	17
9 OS	29 + 3	1180	N	Y	Y	N	N	Y	Laser	N	Y	Y	Z2S3+	37 + 6	N	–	–	Laser, surgery	4b	17

OD = right eye; OS = left eye; GA = gestational age (weeks + days); BW = birth weight (grams); NEC = necrotizing enterocolitis; BT = blood transfusion; BPD = bronchopulmonary dysplasia; PDA = persistent ductus arteriosus; O_2_ = received oxygen therapy; Z = zone; S = stage; + = plus disease; FU = period of follow-up (months); Y = yes; N = no; − = not applicable.

*if no injection was performed.

**Table 2:** Summary of studies on the use of intravitreal ranibizumab for ROP.

Authors	Study design	Sample size	Criteria for treatment with anti-VEGF	Initial regression	Recurrence rate	Subsequent Treatment	Complications
Castellanos *et al.*^22^	Retrospective	6 eyes of 3 infants	Type 1 ROP	100%	0%	–	–
Chen *et al.*^23^	Retrospective	31 eyes of 16 infants(ranibizumab) 41 eyes of 21 infants (bevacizumab)	Type 1 ROP	100% (ranibizumab) 98% (bevacizumab)	0%	Laser	–
Wong *et al.*^29^	Retrospective	6 eyes of 4 infants (ranibizumab) 4 eyes of 2 infants (bevacizumab)	Zone 1 or posterior Zone2 disease	100% (both ranibizumab + bevacizumab)	83% ranibizumab 0% bevacizumab	Laser	–
Erol *et al.*^24^	Retrospective	15 eyes of 8 infants (ranibizumab) 21 eyes of 12 infants (bevacizumab)	Type 1 ROP	100% (both ranibizumab + bevacizumab)	40% ranibizumab 10% bevacizumab	Laser	–
Mota *et al.*^27^	Retrospective	4 eyes of 2 infants	APROP	Case 1: bilateral ranibizumab injection → initial bilateral regression then recurrence at 4 weeks → 2^nd^ injection + laser → regress Case 2: initial laser therapy → disease progression → ranibizumab + supplementary laser → regress			–
Lin *et al.*^26^	Retrospective	2 eyes of 1 infant	Zone 1 Stage 3 Plus	Bilateral bevacizumab injection → initial regression → recurrence at 2 months → laser + ranibizumab → regress			–
Jang *et al.*^28^	Retrospective	2 eyes of 1 infant	Zone 1 Stage 3 Plus	Initial combined bilateral ranibizumab with laser → regress → bilateral recurrence with total retinal detachment at 4 months			–
Present series	Retrospective	10 eyes of 6 infants	Selected cases of Type 1 ROP, eg APROP, poor pupil dilatation	63%* (70%^)	60%* (43%^)	Laser, surgery in 1 case	Non–progressive peripheral lens opacity

^*^Only including eyes with intravitreal ranibizumab as initial therapy.

^^^Also including eyes with laser as initial therapy followed by intravitreal ranibizumab.
